# Complete Genome Sequence of a 2019 Novel Coronavirus (SARS-CoV-2) Strain Causing a COVID-19 Case in Morocco

**DOI:** 10.1128/MRA.00633-20

**Published:** 2020-07-02

**Authors:** Sanaâ Lemriss, Amal Souiri, Narjis Amar, Nabil Lemzaoui, Omar Mestoui, Mohamed Labioui, Nabil Ouaariba, Ayoub Jibjibe, Mahmoud Yartaoui, Mohamed Chahmi, Marouane El Rhouila, Samiha Sellak, Nadia Kandoussi, Saâd El Kabbaj

**Affiliations:** a Department of Biosafety PCL3, Laboratory of Research and Medical Analysis of Gendarmerie Royale, Rabat, Morocco; DOE Joint Genome Institute

## Abstract

Here, we report a complete genome sequence obtained for a novel severe acute respiratory syndrome coronavirus 2 (SARS-CoV-2) strain isolated from a nasopharyngeal swab specimen of a Moroccan patient with coronavirus disease 2019 (COVID-19).

## ANNOUNCEMENT

An epidemic of pneumonia, named coronavirus disease 2019 (COVID-19) by the World Health Organization, emerged in Wuhan, China, in December 2019 and has rapidly spread worldwide (1, 2). The virus causing the pneumonia was sequenced, and it was discovered that it is a strain of betacoronavirus (family *Coronaviridae*, genus *Betacoronavirus*); it was named severe acute respiratory syndrome coronavirus 2 (SARS-CoV-2) by the Coronavirus Study Group of the International Committee on Taxonomy of Viruses (3). In Morocco, more than 7,800 cases have been reported (4). To control the disease, it is important to characterize the viral genome sequences.

A nasopharyngeal swab specimen was collected on 23 April 2020 from a man who had contact with family clusters that tested positive for COVID-19 in Ouarzazate City, Morocco, and was identified as positive for SARS-CoV-2 (threshold cycle [*C_T_*] value, 16.5 in E-gene and 19.48 in RdRp-gene) by real-time reverse transcriptase PCR (RT-PCR) assay as previously reported (5). All participants provided their written informed consent, and the study protocol was conducted according to ethical requirements for human biological research.

Viral RNA of the same sample was extracted using a QIAamp viral RNA minikit (Qiagen). The full genome was amplified according to the CDC protocol (6), with two sets of 38 primers used in two rounds of nested RT-PCR to produce overlapping PCR products covering the full genome. Amplicons were purified with ExoSAP-IT PCR product cleanup (Applied Biosystems) and quantified with a QFX fluorometer (DeNovix) before being pooled at an equimolar concentration. The library was prepared with the Nextera XT library prep kit (Illumina), purified with Agencourt AMPure XP beads (Beckman Coulter), and quantified with a QFX fluorometer. The resulting DNA library was sequenced with a MiSeq system using 250-bp paired ends (Illumina).

The fastq files (139,949 paired-end reads) were cleaned with Trimmomatic 0.36 (TRAILING:35, SLIDINGWINDOW:20) and then subjected to mapping with the reference SARS-CoV-2 genome (GenBank accession number MN908947.3) using GS Reference Mapper 2.9 with default parameters (7), and a consensus sequence named hCoV-19_Morocco_OUA677_19_2020 was obtained, which has an average coverage of 1,000× and 37.97% GC content.

The open reading frames (ORFs) were predicted using Geneious Prime 2020.1 (8) and annotated using the “CD-Search” tool in the Conserved Domain Database with default parameters (9–11). Sequence alignments with the SARS-CoV-2 genome (GenBank accession number MN908947.3) were done with CLC Genomics Workbench 20, using the “create Alignement 1.02” tools with default parameters (12).

The genome sequence is 29,934 nucleotides (nt) long, including a 5′ untranslated region (UTR) (nt 1 to 301), replicase complex ORF1ab (nt 301 to 21590), S gene (nt 21598 to 25419), ORF3a (nt 25428 to 26255), E gene/ORF4 (nt 26280 to 26507), M gene/ORF5 (nt 26558 to 27226), ORF6 (nt 27237 to 27422), ORF7a (nt 27429 to 27794), ORF7b (nt 27791 to 27922), ORF8 gene (nt 27929 to 28294), N gene/ORF9 (nt 28309 to 29568), ORF10 gene (nt 29593 to 29709), and a 3′ UTR (nt 29710 to 29921).

**FIG 1:**
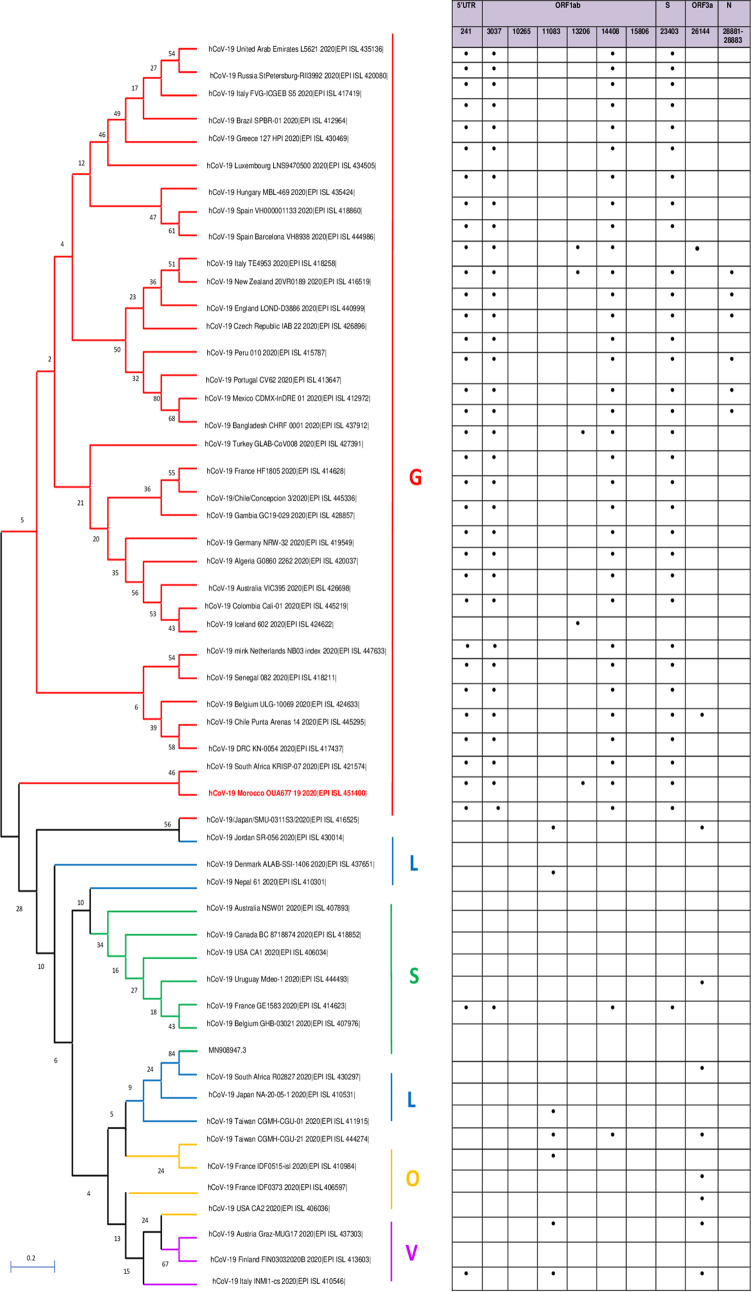
Phylogenetic tree of the complete nucleotide sequence of hCoV-19_Morocco_OUA677_19_2020 and 54 other global strains obtained from the GISAID database, associated with a table representing single-nucleotide polymorphisms (SNPs). The phylogenetic tree was constructed with the neighbor-joining method using MEGAX, and the reliability of each tree branch was estimated by performing 1,000 bootstrap replicates.

Compared with the reference strain (GenBank accession number MN908947.3), the hCoV-19_Morocco_OUA677_19_2020 genome has a total of 10 nucleotide variations. We detected mutations in noncoding positions of G to A (position 198), another one of C to T (position 241), two mutations of A to T (position 29881 to 29882), and other mutations in coding regions that generated amino acid changes, such as F924F, P4715L, S5039I, and L6082F in the polyprotein encoded by the ORF1ab gene, D614G in the spike glycoprotein (S), and A155A in the nucleocapsid protein (N) (Fig. 1).

The single-nucleotide polymorphisms (SNPs) of the Moroccan sequence were further defined from 54 sequences of SARS-CoV-2 representing 50 countries all over the world (Fig. 1). Important substitutions were observed among the different ORFs (ORF1ab, 82; S segment, 33; N segment, 18; ORF3a, 10; noncoding region 5′ UTR, 35).

Phylogenetic analysis of this virus genome compared with 54 selected sequences showed that it was grouped in SARS-CoV-2 clade G, which includes strains from Asia, Europe, North America, Australia, and Africa (Fig. 1).

We are currently sequencing and analyzing more complete genomes from different regions of Morocco to understand the virus dispersion and to associate this information with epidemiological data.

### Data availability.

The consensus data for the hCoV-19_Morocco_OUA677_19_2020 genome have been deposited in the GISAID database (accession number EPI_ISL_451400) and GenBank (accession number MT513758). The accession numbers for the Illumina MiSeq sequence raw reads in the NCBI Sequence Read Archive (SRA) are PRJNA637892 (BioProject), SRR11945456 (SRA), and SAMN15160097 (BioSample).
